# The Role of Ion Channels and Transporters in Human Health and Diseases

**DOI:** 10.3390/biomedicines13122871

**Published:** 2025-11-25

**Authors:** Teresa Soda

**Affiliations:** Department of Health Sciences, University of Magna Graecia, 88100 Catanzaro, Italy; teresa.soda@unicz.it

Ion channels and transporters are increasingly being recognized as key in the maintenance of cellular homeostasis and pivotal contributors to the pathogenesis of a wide range of human diseases. In recent years, accumulating evidence has expanded our understanding of these molecular systems beyond traditional electrophysiological domains, revealing their complex involvement in signaling pathways, metabolic regulation, and cellular plasticity. This concept is reinforced by the extensive literature demonstrating the centrality of ion channels in neuronal excitability and cardiovascular physiology [1,2].

This Special Issue, “The Role of Ion Channels and Transporters in Human Health and Diseases,” gathers a diverse collection of original studies and comprehensive reviews that together outline the multifaceted functions of ion channels and transporters in different physiological systems and their dysfunction in pathological contexts.

Among the most thought-provoking contributions is the review by Brunetti et al. [3], which challenges the canonical view of ionotropic glutamate receptors (iGluRs) as mere ion-conducting proteins. The authors elegantly dissect the growing body of evidence supporting flux-independent (metabotropic-like) signaling by AMPA and NMDA receptors, in agreement with independent studies on non-ionotropic NMDA receptor signaling [4]. These alternative signaling modes have significant implications not only for synaptic plasticity but also for glial function and cerebrovascular physiology, opening the door to novel therapeutic strategies aimed at selectively modulating receptor functions beyond ion permeability.

Equally relevant is the work by Ueno et al. [5], which details the expression and regulatory interactions of transporters, ion channels, and junctional proteins in choroid plexus epithelial cells. Their review underscores how alterations in the blood–cerebrospinal fluid barrier can contribute to neurological dysfunction and highlights a crucial but often overlooked anatomical structure in neurodegenerative and inflammatory disorders.

In the field of cancer biology, recent work highlights the interplay between ion channels and aquaporins in shaping cell volume regulation, proliferation, and apoptosis—concepts consistent with broader evidence on ion channels as oncogenic drivers [6].

The cardiovascular domain is well represented in this Special Issue. Castagna et al. [7] provide an up-to-date and detailed review of the sodium chloride cotransporter (NCC) in hypertension. They explore how genetic, hormonal, and dietary factors influence NCC regulation and how insights from urinary extracellular vesicles may support precision medicine approaches in hypertensive patients.

Bridging cardiovascular and neurological systems, the review by Soda et al. [8] presents a comprehensive synthesis on the heart–brain axis, focusing on how cardiovascular disorders contribute to cognitive decline via impaired synaptic plasticity. Their findings resonate with other investigations into neuro-cardiac interactions and the molecular bases of cardiogenic dementia [9].

Finally, Murasheva et al. [10] explore the neuroprotective effects of SGLT2 inhibitors and GLP-1 receptor agonists in experimental models of stroke, detailing their ability to mitigate both neuronal and glial damage. This suggests that the modulation of metabolic and ionic balance may represent a viable strategy in stroke management, particularly in diabetic patients.

Taken together, the articles in this Special Issue illuminate the centrality of ion channels and transporters in human physiology and disease. They highlight the conceptual shift from viewing these molecules merely as passive conduits for ion flow to appreciating their active role in cellular signaling, plasticity, and pathology. We hope this collection, complemented by broader research in the field [1,2,4,6,9,11], will inspire future studies that aim to decipher the still uncharted roles of ion channels and transporters and translating these findings into innovative diagnostic and therapeutic tools.
